# Supplementary material for: Spatio-temporal breather dynamics in microcomb soliton crystals
Source: Light Sci Appl. 2024 Sep 12;13:251. doi: 10.1038/s41377-024-01573-4 (PMC11393309; doi:10.1038/s41377-024-01573-4)
Supplement: Supplementary file 1 — supplemental material [file 41377_2024_1573_MOESM1_ESM.pdf]

Supplementary Information for
Breather dynamics in microcomb soliton crystals

Futai Hu[1†*], Abhinav Kumar Vinod[1†*], Wenting Wang[1], Hsiao-Hsuan Chin[1], James F. McMillan[1], Ziyu Zhan[2], Yuan Meng[2], Mali Gong[2], and Chee Wei Wong[1*]

[1] Fang Lu Mesoscopic Optics and Quantum Electronics Laboratory, University of California, Los Angeles, CA, USA.

[2] State Key Laboratory of Precision Measurement Technology and Instruments, Tsinghua University, Beijing 100084, China.

[†]These authors contributed equally to this work.

[*]Corresponding authors: phyhft@gmail.com, abhinavkumar@ucla.edu, cheewei.wong@ucla.edu

Supplementary Note 1. Avoided-mode crossings and their temperature dependences

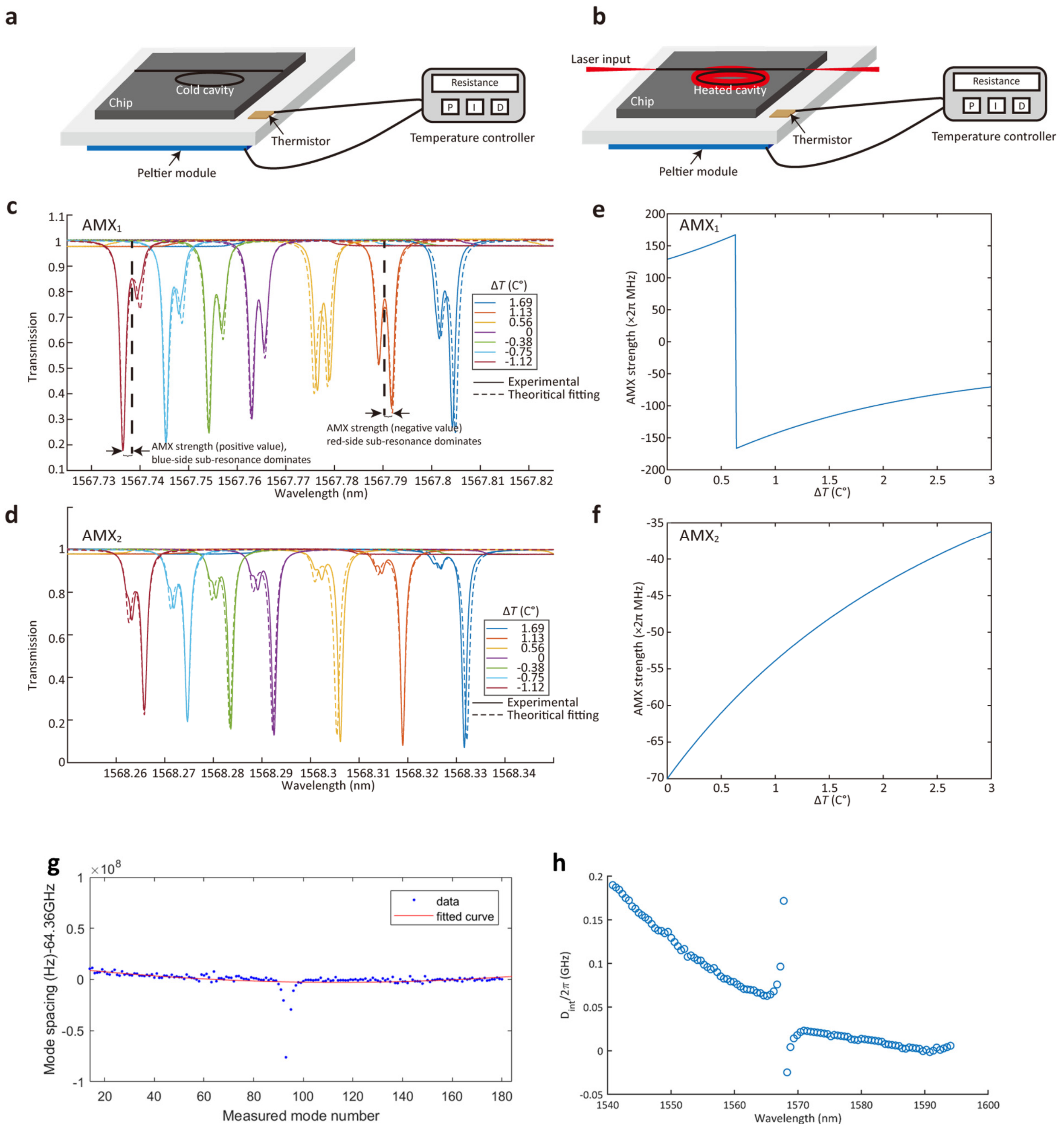

Figure S1 | a, The thermal configuration of the device under test. A Peltier module (CM29-1.9-04AC) is attached with the metal platform that holds the chip. This Peltier module can actively control the chip temperature via a PID feedback provided by a temperature controller (TED200C) connected to a thermistor (USP12837). The microcavity works as a cold cavity when the intracavity power is low. **b,** The microcavity works as a hot cavity when the laser input is coupled

into the microcavity. **c** and **d,** The experimental (solid line) and fitted (dashed line) transmission spectra near AMX_1 (**c**) and AMX_2 (**d**) of the cold cavity at different temperatures. The experimental transmission is normalized to 1, and the experimental data is plotted in solid line because of the high data density. The fitting is based on the coupled-mode theory. The fitting is based on the coupled-mode theory. The unperturbed modal frequency is marked with black dashed lines. The AMX strength is defined as the shifting frequency of the deepest sub-resonance with respect to the unperturbed modal frequency. **e** and **f**, The strengths of AMX_1 (**e**) and AMX_2. (**f**) of the cold cavity at different temperatures. The AMX strength is the frequency shift of the dominant sub-resonance with respect to the resonance without AMX. **g**, The measured and fitted mode spacing as a function of the measured mode number at $\Delta T = 0$°C. **h**, Measured D_{int} as a function of the wavelength.

As shown in Fig. S1a, the microcavity works as a cold cavity when low-power light is coupled into the microcavity. The local temperature near the microresonator T is the same as the chip temperature. Mode indexes of the microresonator are known to be a function of the lattice temperature. When the pump is coupled into the microcavity, the intense intracavity power creates a hot cavity via the photothermal effect, as illustrated in Fig. S1b. T then deviates from the chip temperature, thereby affecting the resonance wavelength and the AMX strength.

One of the significant characteristics of the mode crossing is the resonance splitting near the AMX point. Each resonance near the crossing point splits into two sub-resonances. Here we do not specify the AMX strength as the highest perturbed mode. Instead, each perturbed mode has its AMX strength. The AMX strength is determined by the frequency shift of the deepest sub-resonance with respect to the unperturbed modal frequency before splitting.

Directly imaging AMXs of the hot cavity is possible but difficult. Instead, we simulate the hot cavity by tuning T of the cold cavity. The initial temperature, i.e., the temperature used in comb generation, is set to be 37.03 °C. Adjusting the target resistance of the thermistor through the temperature controller, we can actively induce a temperature variation ΔT. As shown in Fig. S1c,

we firstly compare the transmission spectra near AMX1 of the cold cavity at different ΔT. The hybrid mode caused by AMX has two sub-resonances. At low T, the dominant sub-resonance is the one away from the pump wavelength, creating a positive frequency shift with respect to the resonance without AMX. When we decrease the target resistance of the thermistor to increase the target temperature, the hybrid mode has a red-shifting and the dominant sub-resonance changes to the one close to the pump wavelength. The negative AMX strength corresponds to the locally reduced detuning in the LLE simulation. In contrast, the hybrid mode induced by AMX2 is constantly dominated by the sub-resonance close to the pump wavelength, with the mode resonance approaching a clean resonance without AMX when the temperature increase. Additionally, we experimentally measured the transmission curves of two polarizations and it prove that the AMX type in the current design is polarization-type.

We implement coupled-mode theory to fit the experimental transmission spectra. Assuming that ***a*** and ***b*** are two eigenmodes of the resonator and they have a coupling strength of g, ***a*** and ***b*** are then linked via the coupled-mode theory[1],

$$\begin{aligned}\frac{d\tilde{a}}{dt} &= -(i\omega_a + \gamma_a + \Gamma_a)\tilde{a} - ig\tilde{b} - \kappa_a \tilde{a}_{\text{in}} \\ \frac{d\tilde{b}}{dt} &= -(i\omega_b + \gamma_b + \Gamma_b)\tilde{b} - ig\tilde{a} - \kappa_b \tilde{b}_{\text{in}}\end{aligned} \tag{1}$$

Here, $\tilde{a}$ and $\tilde{b}$ are the amplitude; ω_a and ω_b are the angular mode frequencies near AMX1 at the initial temperature and ω_a-ω_b = δ_{ab}; γ_a and γ_b are the intrinsic loss rates; Γ_a and Γ_b are the coupling loss rate; g is in the unit of Hz. Ignoring the polarization conversion of inside the microresonator, the output and input are related by[1],

$$\begin{aligned}\begin{bmatrix}\tilde{a}_{\text{out}} \\ \tilde{b}_{\text{out}}\end{bmatrix} &= \begin{bmatrix}\kappa_a & 0 \\ \kappa_b & 0\end{bmatrix}\begin{bmatrix}i(\omega_a-\omega)+\gamma_a+\Gamma_a & ig \\ ig & i(\omega_b-\omega)+\gamma_b+\Gamma_b\end{bmatrix}^{-1}\begin{bmatrix}-\kappa_a & 0 \\ -\kappa_b & 0\end{bmatrix}\begin{bmatrix}\tilde{a}_{\text{in}} \\ \tilde{b}_{\text{in}}\end{bmatrix}+\begin{bmatrix}\tilde{a}_{\text{in}} \\ \tilde{b}_{\text{in}}\end{bmatrix} \\ \begin{bmatrix}\tilde{a}_{\text{out}} \\ \tilde{b}_{\text{out}}\end{bmatrix} &= \begin{bmatrix}\sqrt{2\Gamma_a} & 0 \\ \sqrt{2\Gamma_b} & 0\end{bmatrix}\begin{bmatrix}i(\omega_a-\omega)+\gamma_a+\Gamma_a & ig \\ ig & i(\omega_b-\omega)+\gamma_b+\Gamma_b\end{bmatrix}^{-1}\begin{bmatrix}-\sqrt{2\Gamma_a} & 0 \\ -\sqrt{2\Gamma_b} & 0\end{bmatrix}\begin{bmatrix}\tilde{a}_{\text{in}} \\ \tilde{b}_{\text{in}}\end{bmatrix}+\begin{bmatrix}\tilde{a}_{\text{in}} \\ \tilde{b}_{\text{in}}\end{bmatrix}\end{aligned} \tag{2}$$

where *in* and *out* represent the input and output of the field amplitude, respectively. Choosing ***a*** as the primary mode when coupling laser input into the cavity, the transmission is given by $|\tilde{a}_{\text{out}}/\tilde{a}_{\text{in}}|^2$. We use a Particle Swarm Optimization method to find roughly fitting parameters and then manually adjust them to fit the transmission at $\Delta T = 0$. The fitting parameters are listed in Table S1. The coupling strength is larger than the linewidths of both modes.

When taking ΔT into consideration, ω_a and ω_b are expressed as,

$$\begin{aligned}\omega_a(\Delta T) &= \frac{\omega_a(0)}{\text{FSR}_a}(\text{FSR}_a + \frac{d\text{FSR}_a}{dT}\Delta T) \\ \omega_b(\Delta T) &= \frac{\omega_b(0)}{\text{FSR}_a}(\text{FSR}_b + \frac{d\text{FSR}_b}{dT}\Delta T)\end{aligned} \tag{3}$$

We further obtain the fitting value of FSR_b and the thermal dependence of both FSRs.

Table S1 | The parameters used to fit the transmission near AMX1 and AMX2.

Parameters	ω_a	γ_a	γ_b	Γ_a	Γ_b	g	δ_{ab}
Fitting value (×2π MHz)	≈ 1.91×10^8	52	78	30	18	175	94

Parameters	FSR_a- FSR_b	$d\text{FSR}_\text{a}/d\Delta T$	$d\text{FSR}_\text{b}/d\Delta T$
Fitting value	0.45 GHz	-0.155 MHz/C°	-0.146 MHz/C°

Using the fitting parameters in Table S1 and the same temperature sets of experiments, we then obtain the fitted transmission as shown in Figure S1. In general, the experimental result and theoretical fitting match well. The slight spectral mismatch is attributed to the deviation between the actual temperature and estimated temperature via the resistance. The small shoulder in Fig. S1d is caused by a third mode. Here we assume its weak influence on the AMX strength is already considered in mode ***b***. Then we further quantitively analyze the temperature dependence of the AMX strength. Here the AMX strength is defined as the frequency shift of the dominant sub-resonance with respect to the resonance without AMX.

We have observed several interesting features during the generation of soliton crystals: **i**. generation paths are highly repeatable at the dynamical breathing region; **ii**. the transition from 2-

defect breathers to 1-defect breathers; **iii**. the spacing of prominent comb lines increase by one resonator FSR with blue-to-red detuning. **iv**. the spectra envelop near the prominent comb lines. These features can be explained from the perspective of avoided mode crossings (AMX) and the consequent intracavity background wave. **v**. The solitons move in different phases. For example, we attribute features **i**, **ii,** and **iv** to the synergy of AMX at different resonances. Feature **ii** and feature **iii** are a result of background variations, and they are mainly attributed to the temperature dependence of AMX. In Fig. S1e, the change of the sign means that the sub-resonance close to the pump wavelength becomes dominant, enabling Feature **iii**. Then the AMX_1 strength, as well as the AMX_1 strength shown in Fig. S1c, turns weak when the temperature further increases. As discussed later in Supplementary Note 2, the synergies of different AMX_1 allow different states, giving rise to Feature **ii**.

Kerr effect also contributes to the change of mode indexes. Because solitons experience a near-zero effective detuning, the Kerr frequency shift experienced by the soliton can be approximated as the opposite number of the laser detuning. The laser detuning in the simulation is near the level of 0.4 GHz. This value is much smaller than the frequency shift shown in Figures S1e and S1f. Therefore, using the thermal dependence of AMX to explain Feature **ii** and **iii** is sufficient.

Fig. S1g illustrates the measured dispersion curve of the microresonator. The measured dispersion term $D_2/2\pi$ varies to be around 267 kHz, and $D_3/2\pi$ varies to be around -1.2kHz. The dispersion term slightly changes with temperature varying.

Supplementary Note 2. Nonlinear numerical modeling

Lugiato–Lefever equation

The generation and dynamics of soliton crystals in microresonators can be described by a normalized spatial-temporal Lugiato–Lefever equation (LLE) with the second-order and third-order dispersion terms[2-5]:

$$\frac{\partial \psi}{\partial \tau} = -(1+i\alpha)\psi + i|\psi|^2 \psi - \sum_{m=2}^{3} (-i)^{m+1} \frac{\beta_m}{m!} \frac{\partial^m \psi}{\partial \theta^m} + F \tag{4}$$

Here all variables are dimensionless in Eq.(4). ψ is the slowly varying intracavity field amplitude, and F is the external pump amplitude. The square-modulus of F and ψ are normalized to the threshold amplitude for parametric oscillation $F_{\text{threshold}} = \sqrt{\frac{8g\kappa_{ex}}{\kappa^3 \hbar \omega_0}}$, where g is the nonlinearity coefficient, κ is the pumped resonance linewidth (total loss rate), and κ_{ex} is the coupling resonance (coupling loss rate)[3]. α is equal to $2(\omega_0-\omega_p)/\kappa$, describing the detuning between the angular frequency of the pumped resonance ω_0 and the angular frequency of the pump laser. β_m is equal to $-2D_m/\kappa$, where $D_m=\partial^2\omega_\mu/\partial\mu^2|_{\mu=0}$ and μ denotes the number of the resonator mode with respect to the pumped mode. $\psi(\theta, \tau)$ is defined on the intracavity "spatial dimension" θ, and the slow-time "temporal dimension" τ. The azimuthal angle θ ranges from 0 to 2π along the circumference. The normalized time τ is equal to $t\kappa/2$.

The numerical simulations of LLE are conducted through a fourth-order Runge-Kutta in the interaction picture method, which is a split-step Fourier algorithm [2,3]. The total mode number is 1025 in the simulation. The parameters are chosen based on the measured results: FSR = 64.8 GHz, $\kappa/2\pi$ = 188 MHz, $\kappa_{ex}/2\pi$ = 99 MHz, $\omega_0/2\pi$ = 188.11 THz. g is given by $\hbar\omega_0^2 c n_2/n_0^2 V_{\text{eff}}$. To better match simulation and experiment results, we choose $D_2/2\pi$ = 230 kHz and $D_3/2\pi$ = -4 kHz in the simulation. Here $\hbar$ is the reduced Planck constant, $n_2 = 2.5\times10^{-19}$ m^2/W is the nonlinear refractive index, n_0 = 1.98 is the effective mode index, $V_{\text{eff}} = 0.8\times10^3$ μm^3 is the effective mode volume. Notice that the massive simulation is finished before the fine measurement on the microresonator dispersion, so there is a small difference in the parameters in the simulation parameters compared to the experimentally acquired parameters. Unless otherwise stated, these parameters are the default parameters. An additional note for the simulation is that we obtain the intracavity field and corresponding spectra in LLE simulations. In experiments, we obtain the spectra recorded after various losses. Therefore, we phenomenologically add loss after obtaining

the stimulated results to match the experiments. Then we discuss how to simulate soliton crystals and incorporate higher-order dispersion, thermal effect, avoided crossings and noise in our work.

1) Higher-order dispersion

The modal dispersion term is $\sum_{m=2}^{N_D}(-i)^{m+1}\frac{\beta_m}{m!}\frac{\partial^m \psi}{\partial \theta^m}$ in the LLE simulation. N_D determines the highest order in the simulation. In this work, N_D is set to 3.

2) Thermal effect

In the revised manuscript, the thermal effect can be incorporated through the thermo-optics effect. We bridge power dissipation in the microresonator with the cavity FSR, two factors experimentally measurable. Here, we show that these two terms are linearly dependent. The local temperature inside the microresonator is modelled as[2], $\frac{d\delta T}{dt} = -\Gamma_T \delta T + \eta_T U_a$, where the first term describes the thermal relaxation induced by the heat dissipation to environment and the second term represent the thermal heating caused by the intracavity field U_a. The total power dissipation $P_{\text{dissipative}}$, in proportional to U_a, includes intrinsic absorption, scattering and other dissipative processes. The temperature variation results in the change of FSR which is expressed as

$$\delta FSR(t) = -\frac{c}{n_{eff}(t)^2 L}\delta n_{eff}(t) \propto \delta T(t) \propto P_{dissipative}(t)$$
$$\delta FSR(t) = \beta_T P_{dissipative}(t)$$

In simulation, the power dissipation is the product of the damping rate and intracavity field. Then the thermal effect can be directly incorporated as the change of pump detuning and cavity FSR in the simulation.

The dependence between δFSR and $P_{\text{dissipative}}$ can be experimentally retrieved from a cavity resonance measurement using VNA and the power meter. In our measurement, we observed a ≈ 2.28 GHz red-shift at 1569.3 nm with a power dissipation of ≈ 25.5 mW. The FSR variation is calculated to be ≈ 772.9 kHz. We estimate a β_T of ≈ 30.31 MHz/W.

3) Avoided mode crossing

To enable the generation of soliton crystals, we need to consider the avoided mode crossing (AMX) in the simulation. AMX is a result of the strong coupling between different kinds of modes, and it causes local resonance splitting. Here, the impact of resonance splitting is described by a local perturbation on the angular frequency of μth mode $\Delta\omega_{\mu}$, which is also denoted as the AMX strength. In previous literature, the perturbations due to mode crossings is introduced as a modal red-shift near the AMX point[6]. We follow this method in our simulations. This method works well

4) Intracavity noise estimation

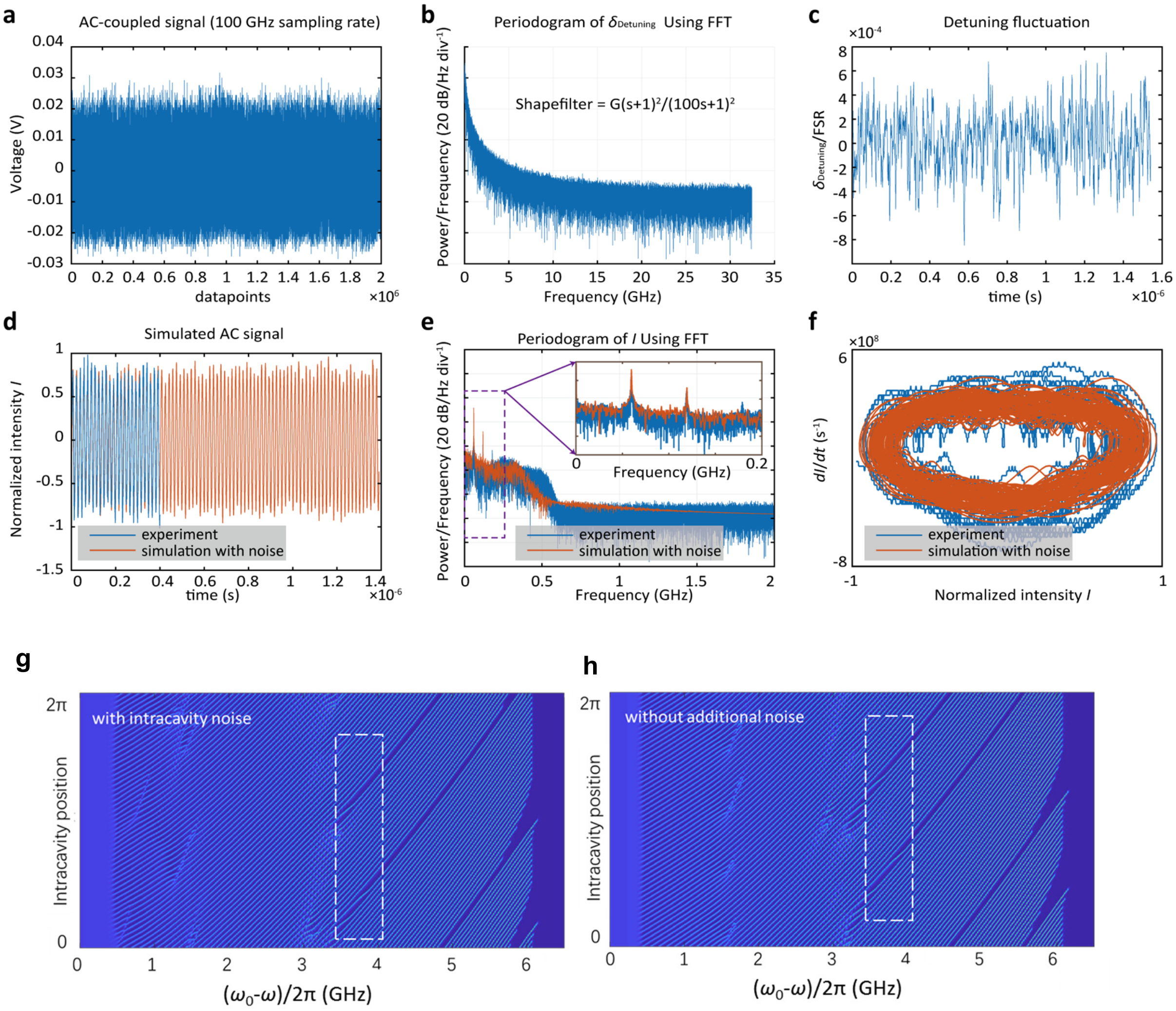

Figure S2 | a, 40 μs-long AC-coupled microcombs signal. **b,** Transfer function we used to manipulate the RF spectral shape of added noise. **c,** Detuning fluctuation with reference to the

signal in **(a)**. **d,** Comparison between simulated and experimental microcombs signals. **e,** RF spectrum of the experimental and simulated AC-coupled signals. The inset is the zoom-in of the RF spectrum with 0-0.2 GHz. **f,** *I-dI/dt* plane to further compare simulated and experimental microcombs signals. (**g, h**) The generation path of soliton crystals, from nonlinear numerical modeling. The dashed rectangles mark where spatial breathers occur. **g,** Simulation with estimated intracavity noise. **h,** Simulation without estimated intracavity noise.

in modeling the breather dynamics in our work. Additional AMX modes are accessible by incorporation of more perturbed modes in simulation. We provide an example in the study of Feature i.

The spatial breathing generally follows a limit cycle in the collective interaction plane, as shown in Fig. 3e of the main text. To study the robustness of spatial breathing with the presence of intracavity noise, we use the AC-coupled microcombs power to analyze the intracavity noise, as plotted in Fig. S2a. For a clear illustration, we attribute the influence of intracavity noise to the fluctuation of detuning. To generate the quasi-random detuning fluctuation that is close to the actual situation, we use a shape-filter function as a low-pass filter applying on the random noise. This process is conducted by ***lsim*** function in MATLAB 2017b. We compare the simulated and experimental AC-coupled microcombs noise and decide the shape-filter function to be $G(s+1)^2/(100s+1)^2$, where s is the variable in ***lsim*** function, and G is the parameter to control peak-to-peak fluctuation. Then we acquire the quasi-random detuning fluctuation as shown in Fig. S2c and consequently obtain the simulated AC-coupled power in Fig. S2d. Fig. S2e shows that the simulated and experimental AC-coupled microcombs noise match well, especially below 200 MHz. We also introduce a new angle to check the matching between the simulated and experimental AC-coupled microcombs noise, as shown in Fig. 2f. The plots in the *I-dI/dt* plane also show the robustness of the spatial breathing. We add the estimated intracavity noise into our simulation on

intracavity patterns and find that we can still obtain a generation path of two-defect breathers. The generation paths with and without estimated intracavity noise are shown in Figs. S2g and S2h.

Stability chat in the detuning-power space

In the following sections, we simulate the stability chart of Kerr combs in the presence of AMX and third-order dispersion. Then we focus on the correspondence between simulations and experimental results of Features **i**, **iv**, and **iv** in main text Figure 2c. The stability chart gives the allowed comb states at different fixed values of the laser detuning and input power. In this section, we consider a negative AMX strength of -60×2π MHz at the 48th mode. To check the allowed stable state, we adopt a common technique [2,6] that seeds the initial intracavity field with a given state and then checks the final states after many roundtrips. Here, we separately use a perfect soliton crystal and a soliton crystal with one defect as the initial state. The soliton spacing of both states is set to be 1/48 of the roundtrip time. We check the final state in the temporal domain and microwave domain after running for 20,000 roundtrips. As shown in Figure S3a, the yellow shade illustrates all allowed states and spatial breathers that exist in the meshed region. Figure S3b presents a deterministic generation path of 1-defect breather and 1-defect soliton crystal.

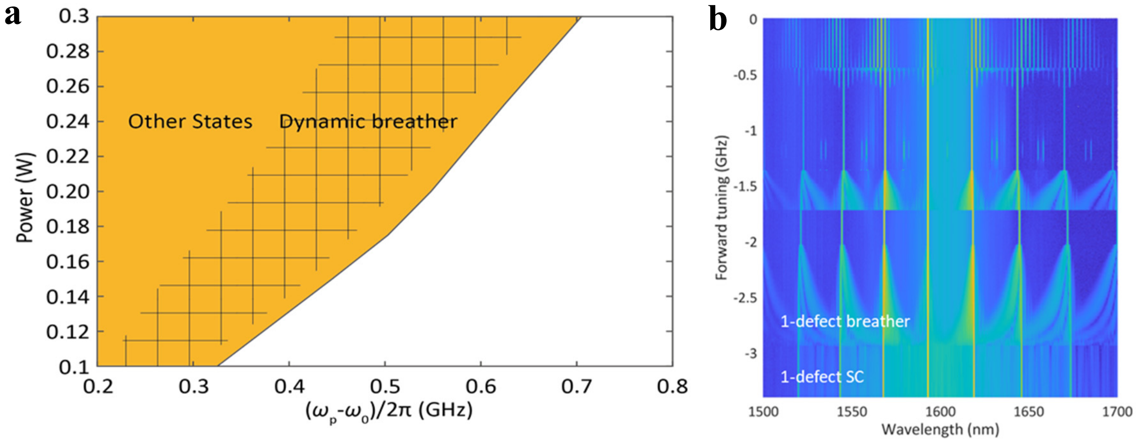

Figure S3 | a, The stability chart of allowed states in detuning-power space. The yellow shade shows the region where frequency combs survive. The spatial breather without much noise can survive in the meshed region. **b,** Experimental optical spectra of generated microcombs from a

64.8 GHz Si3N4 microresonator using forward (blue-to-red) laser sweeping at 25.5 dBm input powers and 1593.1 nm pump wavelength.

Feature i. highly repeatable generation paths & Feature v: The solitons move in different phases

In principle, various soliton crystals may share a similar phase space. However, in the experiment, the generation path is nearly deterministic, where soliton crystal has a fixed defect number and defect spacing. This feature can be attributed to the synergy of AMX at multiple modes, which creates a background wave that allows specific states. To simply illustrate this point, we consider two modes, $\mu_1 = 48$ and $\mu_2 = 47$, with separate AMX strengths. We fix the AMX_1 strength $\Delta\omega_{\mu 1}$ to be -60×2π MHz. The first variable is the AMX_2 strength $\Delta\omega_{\mu 2}$ which takes values of zero, -5×2π MHz and -10×2π MHz. The second variable is the defect spacing of the initial soliton crystal Δd, which ranges from 5/48 to 20/48 of the roundtrip. We run the simulation three times for each ($\Delta\omega_{\mu 2}$, Δd) and check the final state after running for 5 million roundtrips. Fig. S4a and Fig. S4b plot the final intracavity field at $\Delta\omega_{\mu 2} = -5\times2\pi$ MHz, $\Delta d = 10/48$ and $\Delta\omega_{\mu 2} = 0$, $\Delta d = 10/48$. At least one of the moving solitons reaches the peak amplitude in Fig. S4a and Fig. S4b. Apparently, the final states are quite different in the presence of AMX_2. The two movable solitons near the defects no longer move in phase, and their spacing consequently is not an integer time of 1/48. We redefine the dynamic spacing ΔD between two movable solitons as the average of the distance between these two solitons when one of them reaches the amplitude peak. ΔD ranges from 0 to 24/48. The phase difference $\Delta\varphi$ between the two movable solitons ranges from zero to π. As shown in Table S2, the intracavity background wave determined by ($\Delta\omega_{\mu 2}$, Δd) strongly affects the final state (ΔD, $\Delta\varphi$). Most initial states converge to the same stable state (ΔD_1, $\Delta\varphi_1$). The exception marked by grey shade happens when $\Delta d < \Delta D_1$.

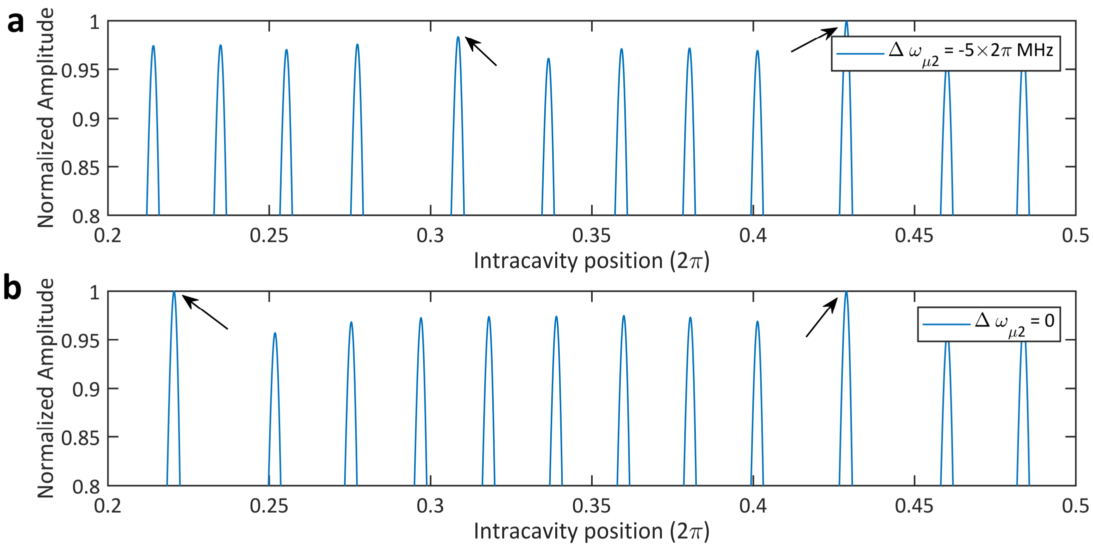

Figure S4 | Normalized intracavity field after running 4 million roundtrips.

Table S2 | The stable state (ΔD, $\Delta\varphi$) at different initial conditions ($\Delta\omega_{\mu2}$, Δd).

$\Delta\omega_{\mu2}$ \ Δd	5/48	10/48	15/48	20/48
0	(3.45/48, 0)	(9.24/48, 0)	(15/48, 0)	(20/48, 0)
-5×2π MHz	(3.39/48, 0.36π)	(6.58/48, 0.34π)	(6.58/48, 0.34π)	(6.58/48, 0.35π)
-10×2π MHz	(3.39/48, 0.30π)	(3.39/48, 0.30π)	(3.39/48, 0.30π)	(3.39/48, 0.30π)

Feature iv. The modified spectra envelope near the prominent comb lines.

The experimental spectrum of stationary soliton crystals with one defect is shown in Fig. S5a. Compared to the simulated spectrum shown in Fig. S5b, it looks quite different, especially near the prominent comb lines. We attribute this difference to multiple AMX points as AMX will introduce a local spectral enhancement (negative AMX strength) or reduction (positive AMX strength). If we consider a bunch of AMX points with respective AMX strengths, the spectrum turns to the one shown in Fig. S5c. The spectral envelope near the prominent comb lines is clearly modified compared to Fig. S5b. The rearrangement of soliton positions induced by dispersion also contributes to the spectral feature in Fig. S5a.

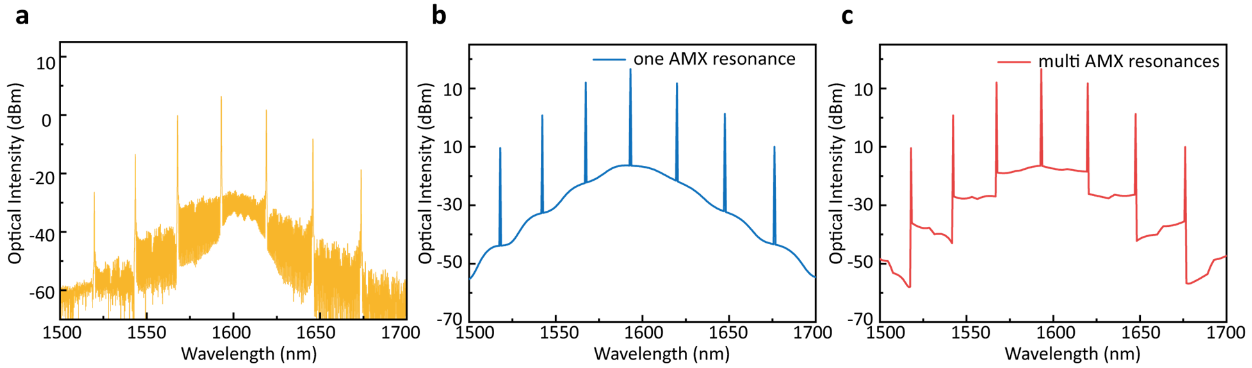

Figure S5 | a, Experimental spectrum of the stationary 1-defect soliton crystal. **b,** Simulated spectral envelope of the stationary 1-defect soliton crystal with one AMX point into consideration. **c,** The modified spectral envelope with multiple AMX points into consideration.

Supplementary Note 3. Supporting numerical results

Bright defect - brief introduction and explanation

In the main text, we mainly discuss the dynamics of dark breathers, i.e., the spatial motion of solitons in soliton crystals with vacancy. Here we provide a brief introduction to the bright defect, i.e., the spatial motion of individual solitons in perfect soliton crystals (PSCs) via elastic collision. Fig. S6a illustrates the collective crossing of two soliton patterns with different group velocities. These two patterns may contain different soliton numbers and have different soliton spacings. The zoom-in of this collective crossing in Fig. S6b shows that the nearly independent crossing of two patterns is accomplished via elastic collisions of solitons. The optical spectrum in Fig. S6b is quite different from the spectra of soliton crystals, and the RF spectrum shows that this state is noisy. Fig. S6c shows the bright breather with two moving individual solitons. Following the above discussion, we still attribute this breather to the collective crossing of two different patterns. In Fig. S6d, pattern 1 turns to be PSC, and pattern 2 turns to be double bounded solitons. This collective behavior can be interpreted as *a soliton moving in a PSC background*. Here we use a previously introduced concept, *meta-soliton*, to name it. There exist prominent lines in the optical spectrum, just like the spectra of other soliton crystals. The RF spectrums show that the intracavity power is still noisy, and there is no breathing peak. We attribute this to the smooth intracavity change in

spatial breathing via elastic collisions. If pattern 2 turns to be a soliton crystal, the collective crossing turns to be a *meta-soliton*. As a result, the spectrum has two kinds of prominent lines with spacings of 36 FSRs and 4 FSRs respectively, which corresponds to two soliton crystals. The RF spectrum has periodic occurring dips.

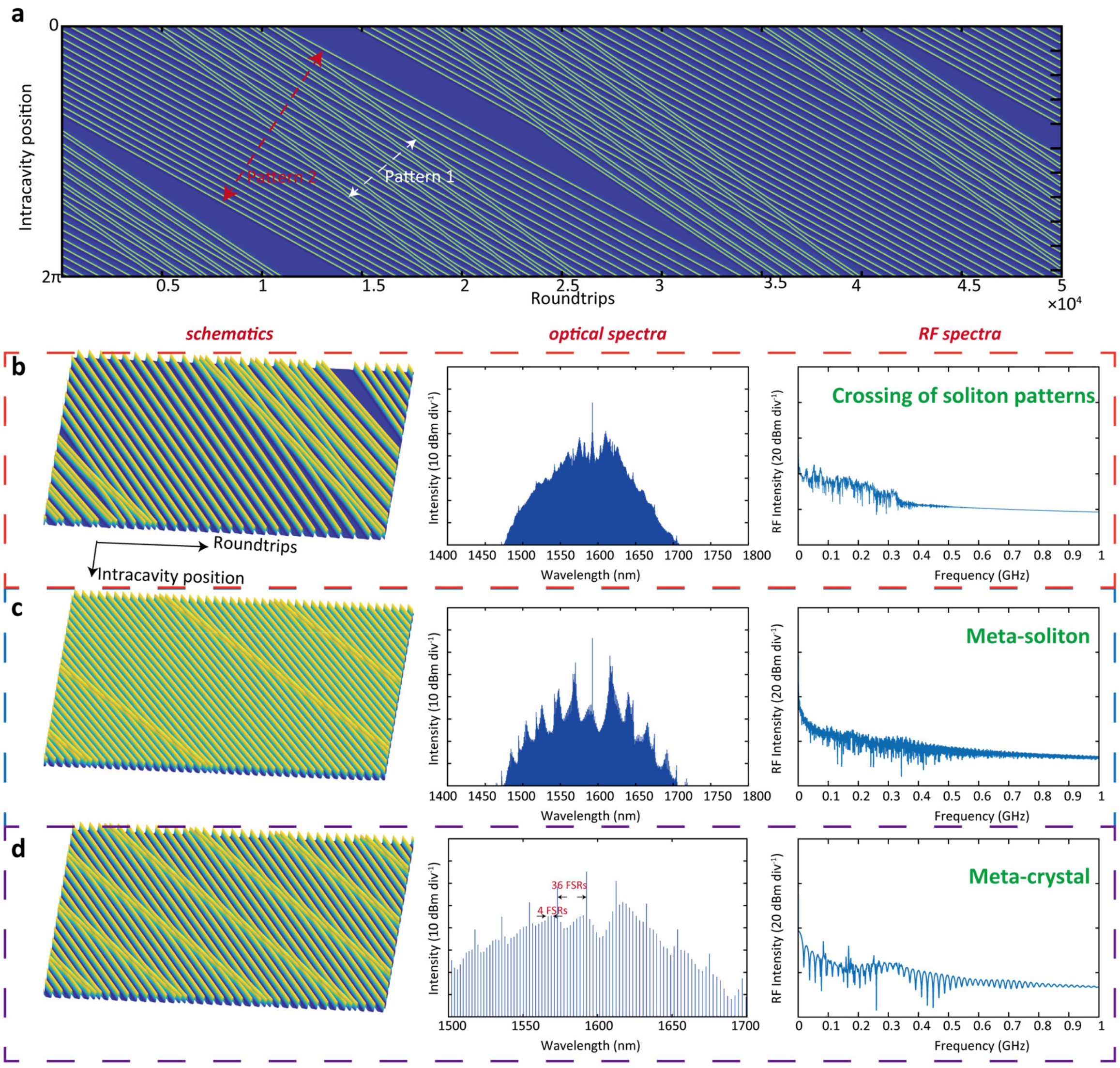

Figure S6 | a, 2D evolution trace of two different patterns with different group velocities. **b,** Schematic, optical spectrum, and RF spectrum of the soliton composites shown in **(a)**. **c,** Schematic, optical spectrum, and RF spectrum of meta-soliton, i.e., bright-defect. **d,** Schematic, optical spectrum, and RF spectrum of meta-crystal, i.e., bright-defect crystal.

Illustration of chaotic soliton waveforms

In the main text, we explain three types of mechanisms that cause chaotic waveforms. In Figure S7, we plot their schematics, optical spectra, and RF spectra.

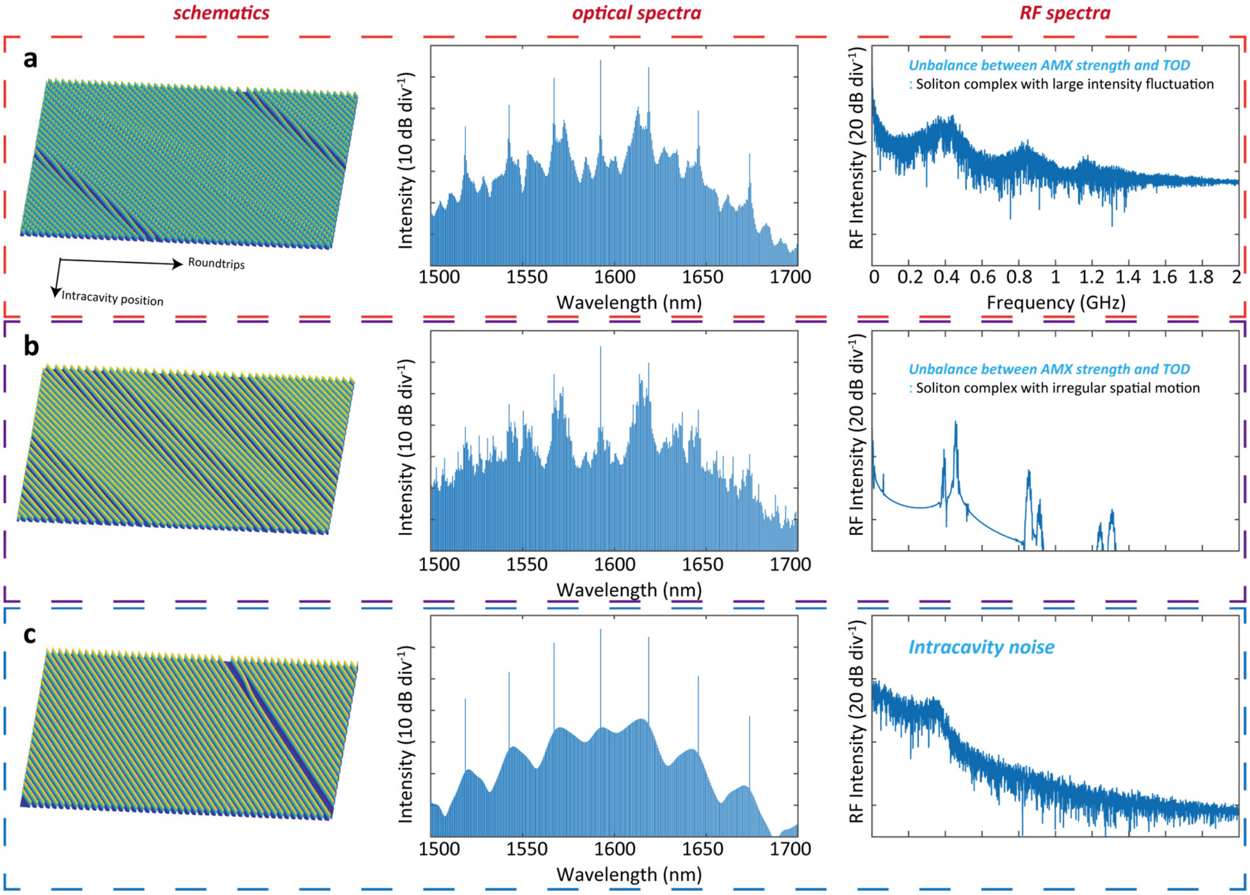

Figure S7 | Three types of chaotic waveforms. **a-b,** Chaotic waveforms caused by the unbalance between the AMX strength and TOD. **a,** Spatial breathers mixed with dense wavy undulations of the soliton intensity. The mixed breather has a high noise level below 2 GHz. **b,** Irregular soliton spatial motion and an uneven spectral envelope. The RF spectrum, however, only becomes noisy at some RF bands. **c,** Chaotic waveforms caused by the intracavity noise. The RF noise mainly comes from the fluctuation of intracavity intensity fluctuation. This chaotic waveform has a relatively smooth spectral envelope. The RF noise mainly occurs at low frequencies.

Supplementary Note 4. Supporting experimental results

Auto-correlation measurement

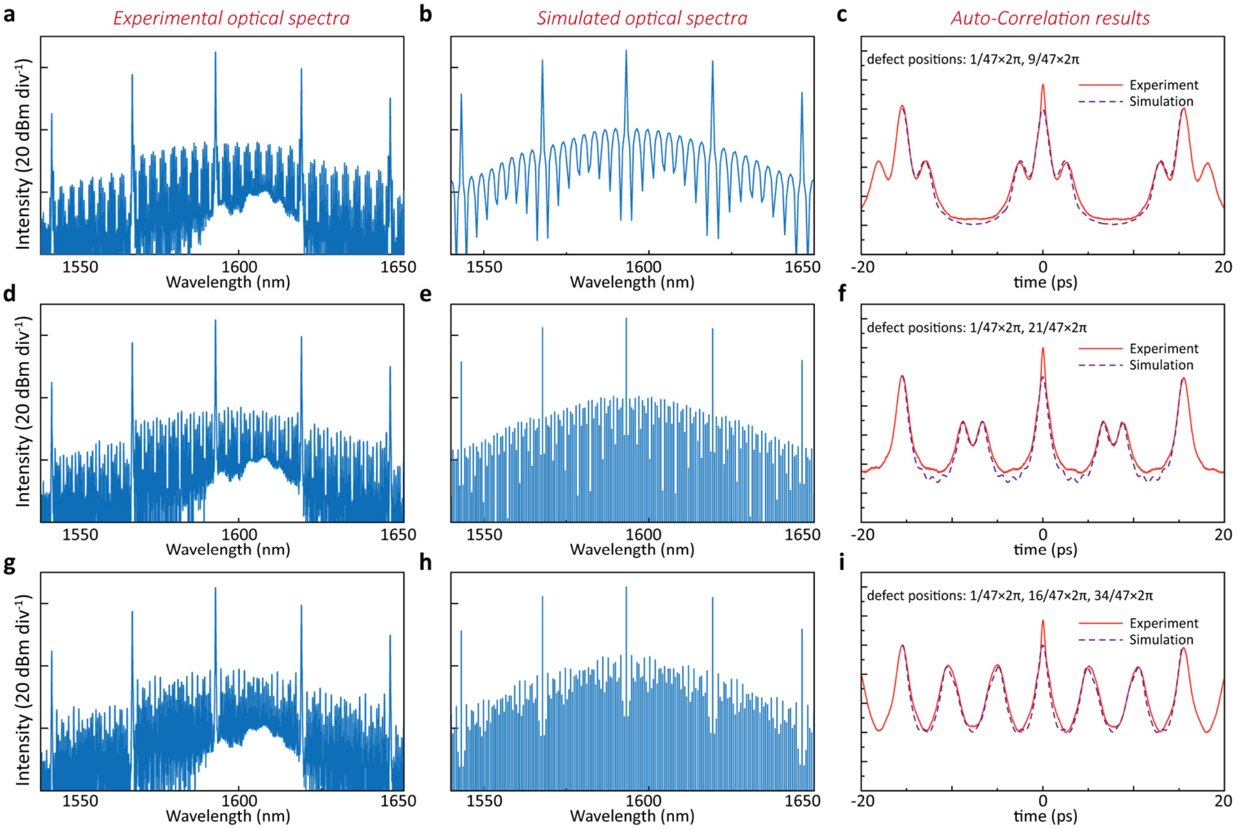

Figure S8 | Experimental optical spectrum (**a**, **d**, **g**), simulated optical spectral envelope (**b**, **e**, **h**), and experimental together with simulated auto-correlation (**c**, **f**, **i**) results for stationary soliton crystals with defects at different intracavity positions. Compared to the other main peaks, the higher intensity of the experimental peak at time = 0 ps is mainly caused by the amplified spontaneous emission from the EDFA in the auto-correlation set-up.

Here we show the method to identify and confirm a soliton crystal with vacancies. The single vacancy shown in Figure S4 is easy to distinguish from other states. For double vacancies, if spectra have a clear pattern like Fig. S8a, we can directly count the arch-type pattern and determine what the state is. In Fig. S8a, we have seven arch-type patterns between two prominent lines, so the relative intracavity positions of two vacancies are determined to be 1/47 ×2π and (1+**7**+1)/47 ×2π. The simulated spectrum confirms our analysis. For a wider spacing or larger vacancy numbers,

this method is not applicable and one needs to generate the simulated spectra, finding the matching spectrum with our experiments. The initial identification can be further checked by auto-correlation results. We use a band-pass filter to filter out the prominent line, and the remaining frequency comb provides direct information about defects, as we discussed in Fig. 3c of the main text. Fig. S8c, Fig. S8f, and Fig. S8i show the experimental and simulated auto-correlation results. These plots help to identify the states.

Origin of AMX

For the supplementary experiment, we used one device with the same geometry design as the one we previously used in the manuscript. A simple schematic setup is shown in Fig. S9a and Fig. S9b. We use a polarization beam splitter to polarize the input light to be TE. Then TM polarization is obtained via a half-wave plate as shown in Fig. S9b. We present the measured transmission curves of two polarizations in Fig. S9c. We only observed one AMX point near 1577 nm within the wavelength range from 1500 nm to 1600 nm. The AMX point of this device red-shifts about 10 nm compared to the AMX point of the previous device. Two transmission curves form good correspondence, examining that the origin of AMX is the polarization. The slight mismatch between transmission curves of TE and TM is attributed to the precision of wavelength information returned from the Santec laser during the sweep.

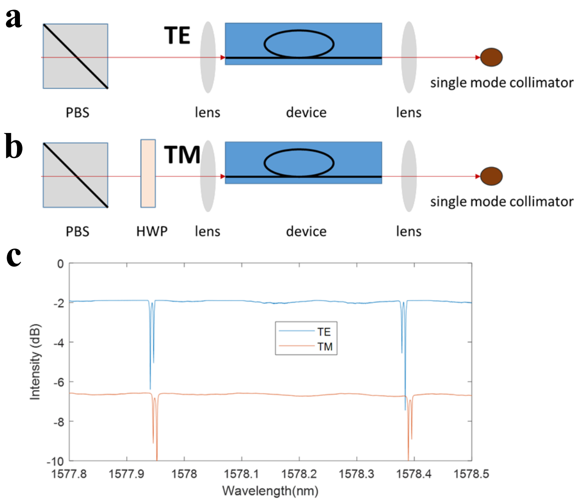

Figure S9 | a, Experimental set-up used for transmission measurement of TE mode. PBS:

polarization beam splitter. **b,** Experimental set-up used for transmission measurement of TM mode. HWP: half-wave plate. **c,** The measured transmission curves of TE and TM modes near the AMX point.

Supplementary References

1 Weng, W. L. & Luiten, A. N. Mode-interactions and polarization conversion in a crystalline microresonator. *Opt. Lett.* **40**, 5431-5434, (2015).

2 Cole, D. C., Lamb, E. S., Del'Haye, P., Diddams, S. A. & Papp, S. B. Soliton crystals in Kerr resonators. *Nat. Photonics* **11**, 671-676, (2017).

3 Godey, C., Balakireva, I. V., Coillet, A. & Chembo, Y. K. Stability analysis of the spatiotemporal Lugiato-Lefever model for Kerr optical frequency combs in the anomalous and normal dispersion regimes. *Phys. Rev. A* **89**, 063814, (2014).

4 Chembo, Y. K. & Menyuk, C. R. Spatiotemporal Lugiato-Lefever formalism for Kerr-comb generation in whispering-gallery-mode resonators. *Phys. Rev. A* **87**, 053852, (2013).

5 Parra-Rivas, P., Knobloch, E., Gomila, D. & Gelens, L. Dark solitons in the Lugiato-Lefever equation with normal dispersion. *Phys. Rev. A* **93**, 063839, (2016).

6 Karpov, M. *et al.* Dynamics of soliton crystals in optical microresonators. *Nat. Phys.* **15**, 1071-1077, (2019).
